# Brain and heart‐specific death in cancer patients: Population‐based study

**DOI:** 10.1002/cam4.4069

**Published:** 2021-08-10

**Authors:** Mohammed Safi, Murad Al‐Nusaif, Dario Trapani, Mubarak A Mashrah, Ravindran Kanesvaran, Aziz Alzandani, Mahmoud Al‐Azab, Syed A Mazher, Abdullah Al‐Danakh, Jiwei Liu

**Affiliations:** ^1^ Department of Oncology First Affiliated Hospital of Dalian Medical University Dalian China; ^2^ Department of Neurology Liaoning Provincial Key Laboratory for Research on the Pathogenic Mechanisms of Neurological Diseases, First Affiliated Hospital, Dalian Medical University; ^3^ IEO ‐ Istituto Europeo di Oncologia Milan, IRCCS Milan Italy; ^4^ Guangzhou Institute of Oral Disease Stomatology Hospital of Guangzhou Medical University, Guangzhou Guangdong China; ^5^ National Cancer Center Singapore; ^6^ Guangzhou Women and Children's Medical Center, Guangzhou Medical University Guangzhou 510623 China; ^7^ Faculty of Medicine and Health Sciences, Thamar University; ^8^ Division of Hematology/ Oncology, UT Southwestern, Clements University Hospital 6201 Harry Hines Blvd Dallas Texas 75390; ^9^ Department of Urology First Affiliated Hospital of Dalian Medical University

**Keywords:** brain metastases, brain–heart axis, epidemiology, heart block, lung cancer

## Abstract

**Background:**

The occurrence of cardiovascular events is a major cause of death in patients with cancer. Small studies have documented a connection between specific brain alterations and autonomic cardiac dysfunctions, possibly resulting in a worse prognosis. We aimed to refine the knowledge of fatal cardiac events in patients with brain metastasis (BM).

**Methods:**

We performed a Surveillance, Epidemiology, and End Results SEER registry‐based investigation (timeline: 2010–2016) and extracted all the advanced patients who had experienced fatal cardiac outcomes. Populations were compared according to the presence or not BM. Kaplan–Meier (KM) methodology was used for survival analysis and a multivariate model was developed by adjusting for multiple possible confounders.

**Results:**

Most related BM and cardiac death were observed at the site of lung cancer (81.4%). We extracted 3187 patients with lung cancer site, including 417 patients who had experienced fatal heart‐specific with a history of BM, which is considered a BM group. The second group of heart‐specific death included 2770 patients was stated as a non‐BM group. Patients who had experienced heart‐specific death in the BM group were predominately male, right side, upper site, and non‐small type (62.11%, 54.92%, 51.56%, 69.78%), respectively. The survival outcomes between BM and the non‐ BM was significantly prominent (*p* = 0.003; median: 2 months vs. 3 months).The negative prognostic independent significance of heart‐fatal events was confirmed after adjusting for multiple variables (HR = 0.76, CI = 0.68–84, *p* < 0.0001). The metastatic liver site was significantly associated with poorer survival rates (HR = 0.68; CI = 0.52–0.88, *p* = 0.005). We revealed a possible connection between the brain and heart functions.

**Conclusions:**

The prognosis of heart‐specific death patients in BM is unfavorable compared to non‐BM settings in lung cancer. We may be at the gates of a new field of neurocardiooncology.

## Graphical abstract

Various studies reported the relation of brain incidents and associated heart dysfunction but no in cancer patients. This first study reported the association between brain metastasis and related lower survival in heat‐related mortality in cancer patients. We uncovered that the associated heart‐specific death and brain metastasis has been occurred mostly occurs in the lung cancer type among all cancer types. We might open a new era for a new field of neuro‐cardio‐oncology. However, the specific mechanisms of pathogenesis warrant further research in the cancer field.

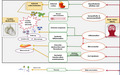

## 1 INTRODUCTION

An increasing body of evidence points to a functional connection between cerebral vascular injuries and damaged cardiac functions, which has been proposed as another cause of heart dysfunction in patients with neurological changes.^1^ Although data for the role of pre‐existing cardiac conditions in raising the likelihood of ischemic stroke is being consolidated, evidence for cerebral ischemic events that can lead to abnormalities of heart function has been identified.^2^ However, there is no clear evidence of the perception of other brain disorders, including malignancies, causing cardiac changes.

Brain metastasis (BM) is crippling and potentially life‐threatening diseases and commonly affects detrimentally patient's prognosis and quality of life. BM is widespread in patients with lung cancer, accounting for up to 40% of cases in the advanced stage.^3^, ^4^ The treatment of BM in lung cancer has had some effect on the outcome, whether with whole‐brain radiation therapy (WBRT), stereotactic radiosurgery (SRS), or SRS‐accompanied surgical resection.^5^ Although significant advances have been made in cancer management in recent years, the prognosis for BM patients is still poor.^6^

The therapeutic and preventative approaches for progressive atherosclerosis are now ready to be considered a popular underline of illness as a population finding in ischemic syndromes and clinical practice. The interconnection between heart and brain has been studied in several disease models of non‐ischemic brain disorders, such as Alzheimer's disease (AD),^7^ demonstrating the concept of non‐atherosclerosis pathways of heart‐brain interrelationship. As a result, we questioned if the presence of BM could impair particular autonomic central functions and affect cardiovascular morbidity and mortality, leading to patients’ poor prognosis in this setting. In order to answer this issue, we designed the first research in the field, which looked at the rate of fatal cardiac events in cancer patients with and without BM, as well as the associated survival.

## 2 PATIENT AND METHODS

The ethical statement is given permission to the SEER study data files by using the reference number 19916‐Nov2019. The SEER database details are not subject to informed patient consent. SEER 18 registries 2019 patients were marked with additional treatment fields using SEER* Stat software (version 8.3.8). The SEER program of the National Cancer Institute is responsible for the collection and reporting of cancer incidence and survival data from several populations on the basis of central cancer registries that cover approximately 30% of the U.S. population. The SEER data include patient demographic information, primary tumor site, tumor morphology, stage at diagnosis, first course of cancer treatment, and follow‐up for vital status. First, collect all cancer sites (Site recode ICD‐O‐3/WHO 2008) with years interval (2010–2016) with stage IV Adult patients’ data were collected from the SEER public database based on the 2019 submission; the incidence data with additional treatment fields were included. The majority of associated BM and heart‐specific death was seen in lung cancer site (81.4%) Supporting Information File 1. Then, we extracted data on patients with Stage IV Lung and bronchus (Site recode ICD‐O‐3/WHO 2008) with a period between 2010 and 2016 and divided the population into two groups: BM group and non‐BM group.

Only patients with active follow‐up during and after treatments were included to minimize the missing data. The following variables were selected: age (20 years or more), sex (male or female), tumors subtype (based on the ICD‐O‐3 convention from the International Classification of Diseases for Oncology—Third Edition, considering only invasive tumors), Histobehave (non‐small type, small type, others), tumors grading (I–II, III–IV, unknown), race (Black, White, or others), primary site labelled (upper, lower, or others), metastatic sites (bone, liver, or lung), receipt of radiation treatment or chemotherapy treatment, laterality (right, left, or others), marital and insurance status. We used the Strengthening the Reporting of Observational Studies in Epidemiology (STROBE) guidelines^8^ to conduct the investigation.

The baseline demographics of patients were compared using the chi‐squared test and *t*‐test for categorical or continuous variables. We analyzed the survival curves with the KM method; the survival curves were compared with the log‐rank test and Cox proportional hazard model for multivariate analysis. Significance was set at *p* < 0.05. Graphical abstract was provided to further explain pathways of brain‐heart dysfunction interactions, according to the literature.

## 3 RESULTS

We extracted 3187 patients with lung cancer from SEER (timeline: 2010–2016), including 417 patients who had experienced fatal heart‐specific disease with a history of BM, which is considered as a BM group. The second group of heart‐specific death included 2770 patients was stated as a non‐BM group. Patients who had experienced heart‐specific death in the BM group was predominately male, right side, upper site, and non‐small type (62.11%, 54.92%, 51.56%, 69.78%), respectively. Detailed other patients characteristics were summarized in Table 1.

**TABLE 1:** Lung cancer patients’ characteristics

Parameters	BM (*N* = 417)	Non‐BM (*N* = 2770)	
Age			0.0001
20–64	149 (35.73)	535 (19.31)	
65–74	168 (40.29)	845 (30.51)	
>74	100 (23.98)	1390 (50.18)	
Sex			0.396
Male	259 (62.11)	1660 (59.93)	
Female	158 (37.89)	1110 (40.07)	
Race			0.260
White	318 (76.26)	2179 (78.66)	
Black	65 (15.59)	422 (15.23)	
Others	34 (0.08)	169 (6.1)	
Marital status			0.193
Yes	191 (45.8)	1175 (42.42)	
Others	226 (493.41)	1595 (57.58)	
Grade			0.001
I–II	35 (8.39)	307 (11.08)	
III–IV	119 (28.54)	569 (20.54)	
Unknown	263 (63)	1894 (68.38)	
Origin			0.807
Right	229 (54.92)	1489 (53.93)	
Left	160 (38.37)	1101 (39.88)	
Others	28 (0.07)	171 (6.19)	
Mets site			
Lung			0.05
Yes	88 (21.1)	708 (25.6)	
No	329 (78.9)	2062 (74.4)	
Bone			0.000
Yes	122 (29.3)	584 (21.1)	
No	295 (70.7)	2186 (78.9)	
Liver		.	0.370
Yes	67 (16.1)	399 (14.4)	
No	350 (83.9)	2371 (85.6)	
Primary site labeled			0.250
Upper	215 (51.56)	1295 (46.75)	
Lower	90 (21.58)	662 (23.9)	
Others	112 (0.27)	813 (29.35)	
Histology			0.044
Non‐small cell lung cancer	291 (69.78)	1787 (64.51)	
Small cell lung cancer	52 (12.47)	342 (12.35)	
Others	74 (0.18)	641 (23.14)	
Radiation status			0.000
Yes	250 (59.95)	695 (25.09)	
No	167 (40.05)	2075 (74.91)	
Surgery			0.339
Yes	12 (2.88)	106 (3.83)	
No	405 (97.12)	2664 (96.17)	
Chemotherapy			0.288
Yes	159 (38.13)	982 (35.45)	
No	258 (61.87)	1788 (64.55)	
Insurance			0.002
Yes	301 (72.18)	2189 (79.03)	
Others	116 (27.82)	581 (20.97)	

**FIGURE 1:**
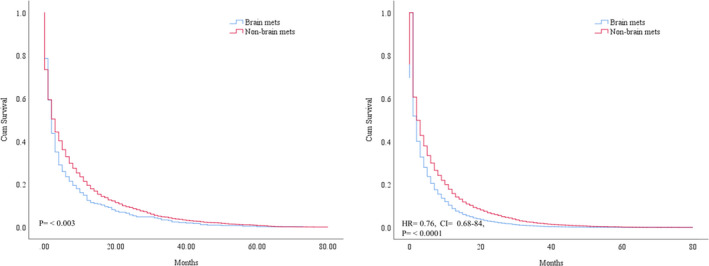
A, KM curve difference comparing heart‐specific death patients in the BM group and non‐BM group (*p* = 0.003). B, Cox multivariate survival with adjusting data between the heart‐specific and overall survival groups (HR = 0.76, CI = 0.68–84, *p* < 0.0001)

**FIGURE 2:**
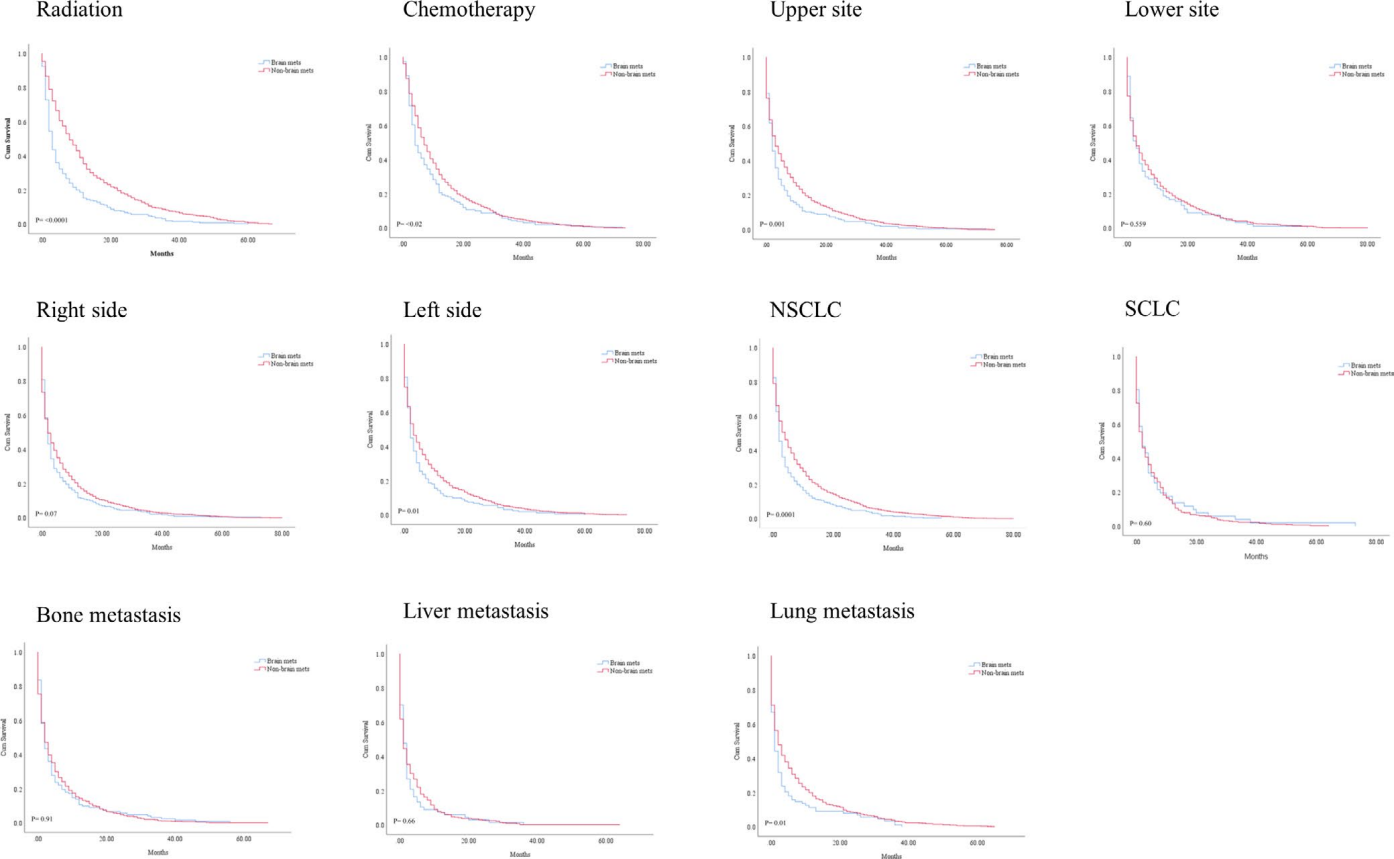
A, KM curve showing OS difference in heart‐specific death patients between the BM and non‐BM groups

**FIGURE 3:**
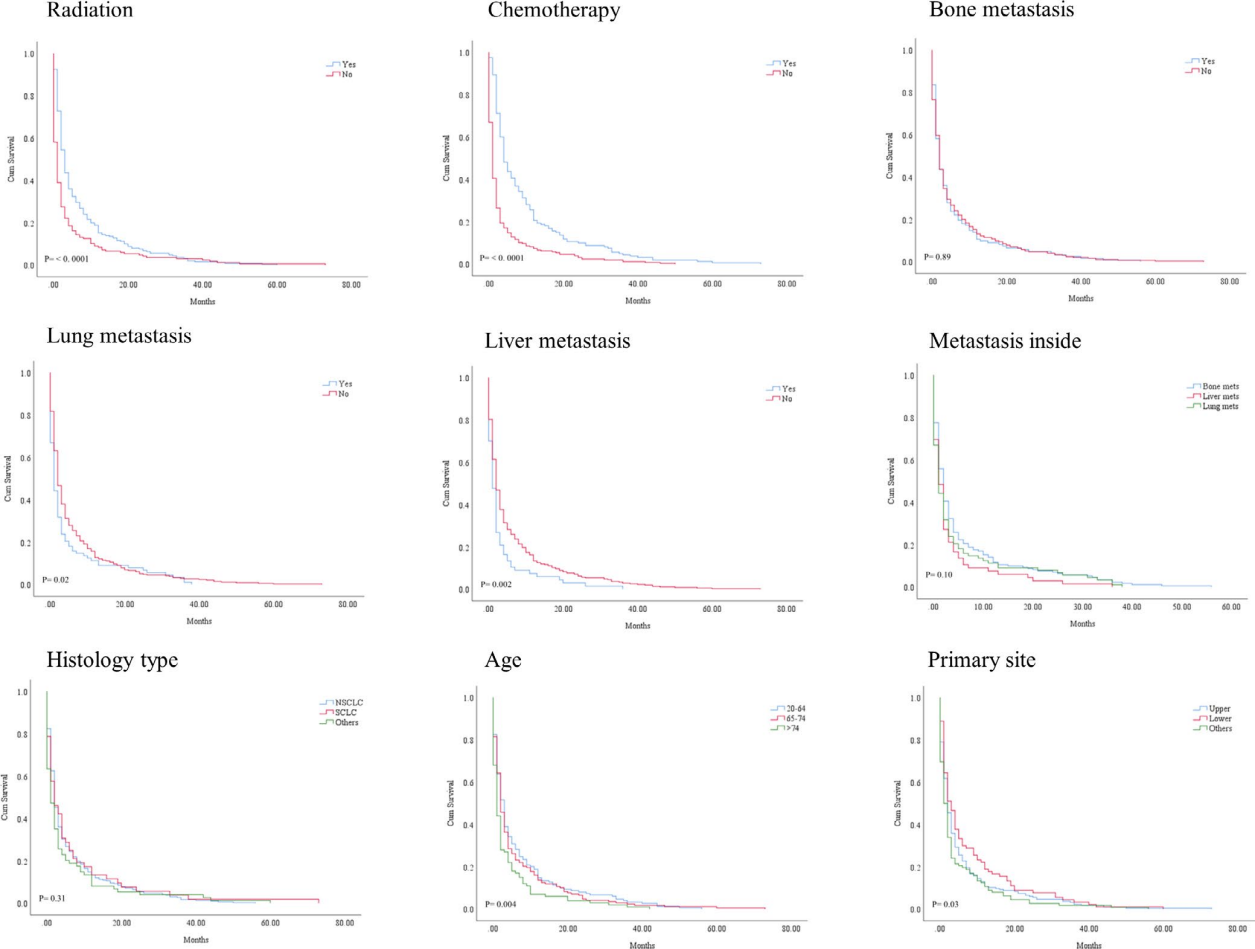
KM curve showing OS difference in heart‐specific death patients of the BM group

The survival outcomes between BM and the non‐ BM was significant (*p* = 0.003; median survival: 2 months vs. 3 months). The negative prognostic independent significance of heart‐fatal events was confirmed after adjusting for multiple variables (HR = 0.76, CI = 0.68–84, *p* < 0.0001). Figure 1. The KM analysis showed a possible predictive value of multiple variables. However, we identified the drivers of the negative prognosis, mainly attributable to older (>74) male gender, white race with upper site location and left side in heart‐specific patients with BM (<0.05). Besides the metastatic pattern to the lung, NSCLC type was negatively associated with poor survival in BM group (*p* = 0.0001, Figure 2, Table 2). The survival effect of each variable inside BM group is shown in Figure 3. In the study inside the BM group, the multivariate analysis confirmed that the metastatic liver site was significantly associated with poorer survival rates (HR = 0.68; CI = 0.52–0.88, *p *= 0.005). Both treatment modality administration modalities (chemotherapy or radiation) were associated with improved survival based multivariate analyses in the BM group (HR = 1.27, CI = 1.03–1.56, *p* = 0.02; HR = 1.86, CI = 1.49–2.31, *p* < 0.0001) respectively Table 3.

**TABLE 2:** KM curve difference comparing heart‐specific death patients in the BM group and non‐BM group

Parameters	BM median (months)	Non‐BM median (months)	Log rank
Age			
20–64	3	4	0.017
65–74	2	3	0.010
>74	1	2	0.007
Sex			
Male	2	2	0.019
Female	2	3	0.067
Race			
White	2	3	0.010
Black	2	3	0.053
Others	2	2	0.945
Marital status			
Yes	2	3	0.009
Others	2	2	0.083
Grade			
I–II	2	6	0.001
III–IV	2	4	0.001
Unknown	2	2	0.683
Origin			
Right	2	2	0.072
Left	2	3	0.018
Others	0	1	0.481
Mets site			
Lung			
Yes	1	2	0.015
No	2	3	0.024
Bone			
Yes	2	2	0.914
No	2	3	0.003
Liver			
Yes	1	1	0.660
No	2	3	0.004
Primary site labeled			
Upper	2	3	0.001
Lower	3	3	0.559
Others	1	1	0.235
Histology			
Non‐small cell lung cancer	2	4	0.0001
Small cell lung cancer	2	2	0.605
Others	1	1	0.918
Radiation status			
Yes	3	8	0.0001
No	1	2	0.006
Surgery			
Yes	5	16	0.412
No	2	2	0.006
Chemotherapy			
Yes	4	7	0.025
No	1	1	0.010
Insurance			
Yes	2	3	0.002
Others	2	2	0.573

**TABLE 3:** Univariate and multivariate analysis of the BM and non‐BM groups

Parameters	BM				Non‐BM			
	Univariate HR (CI)	*p* value	Multivariate HR (CI)	*p* value	Univariate HR (CI)	*p* value	Multivariate HR (CI)	*p* value
Age								
20–64	Reference	0.013		0.34	Reference	0.000		0.142
65–74	1.05 (0.84–1.32)	0.613	1.01 (0.81–1.27)	0.9	1.05 (0.94–1.17)	0.31	1.02 (0.9–1.14)	0.66
>74	1.44 (1.11–1.86)	0.005	1.19 (0.92–1.55)	0.17	1.29 (1.16–1.42)	0.000	1.09 (0.1.9 −1.2)	0.08
Sex								
Male vs. Female	0.962 (0.79–1.31)	0.450			0.91 (0.85–0.99)	0.02	0.86 (0.79‐.0.93)	0.000
Race								
White		0.63				0.505		
Black	1.08 (0.76–1.54)	0.65			1.104 (0.94–1.16)	0.36		
Others	1.2 (0.79–1.83)	0.37			1.06 (0.91–1.25)	0.40		
Marital status								
Yes vs. others	1.01 (0.83–1.23)	0.87			0.93 (0.87–1.01)	0.096		
Grade								
I–II	Reference	0.97				0.000		0.005
III–IV	0.97 (0.66–1.42)	0.89			1.26 (1.1–1.45)	0.001	1.2 (1.09–1.4)	0.002
Unknown	0.96 (0.67–1.36)	0.82			1.55 (1.37–1.75)	0.000	1.2 (1.06–1.38)	0.004
Origin								
Right	Reference	0.75				0.06		0.02
Left	0.97 (0.79–1.18)	0.78			0.92 (0.85–0.99)	0.04	0.92 (−0.85–1.00)	0.06
Others	1.13 (0.76–1.680)	0.53			1.07 (0.91–25)	0.40	0.81 (0.68–0.97)	0.02
Mets site								
Lung	0.78 (0.62–99)	0.054			0.93 (0.85–1.01)	0.116		
Yes vs. No								
Bone	0.98 (0.79–1.22)	0.90			0.83 (0.76–0.915)	0.000	0.7 (0.70–0.85)	0.000
Yes vs. No								
Liver	0.69 (0.53–90)	0.007	0.68 (0.52–0.88)	0.005	0.64 (0.58–0.71)	0.000	0.7 (0.7–0.8)	0.000
Yes vs. No								
Primary site labeled								
Upper	Reference	0.05				0.000		0.017
Lower	0.84 (0.66–1.08)	0.185			0.98 (0.89–1.07)	0.68	0.9 (0.8–1.8)	0.50
Others	1.18 (0.94–1.49)	0.144			1.27 (1.16–1.39)	0.000	1.1 (1.02–1.2)	0.016
Histology								
Non‐small cell lung cancer	Reference	0.39				0.000		0.000
Small cell lung cancer	0.94 (0.70–1.27)	0.71			1.47 (1.15–1.45)	0.000	1.3 (1.2–1.5)	0.000
Others	1.17 (90–1.51)	0.22			1.47 (1.34–1.60)	0.000	1.07 (0.9–1.19)	0.13
Radiation status								
Yes vs. no	1.54 (1.26–1.87)	0.000	1.27 (1.03–1.56)	0.02	1.77 (1.62–1.93)	0.000	1.5 (1.4–1.7)	0.000
Surgery								
Yes vs. no	2.24 (1.18–4.26)	0.013	1.88 (0.98–3.6)	0.05	2.27 (1.8–2.77)	0.000	2.2 (1.8–2.8)	0.000
Chemotherapy								
Yes vs. no	2.05 (1.67–2.51)	0.0000	1.86 (1.49–2.31)	0.000	1.77 (1.64–1.92)	0.000	1.7 (1.5–1.9)	0.000
Insurance								
Yes vs. others	0.96 (0.77–1.19)	0.71			1.09 (1.001–1.20)	0.047		1.00

## 4 DISCUSSION

The function of location and lateralization of brain lesions, clinical biomarkers, and manifestations of cardiac complications, and underlying mechanisms for brain‐heart interaction were discussed in the literature.^9^, ^10^ Neurocardiology has emerged as a discipline that deals with how the brain and the heart interact: the effects of heart damage on the brain and brain damage on the heart.^11^, ^12^ Byer et al. stated for the first time that cerebral vascular damage could cause myocardial damage.^13^ The sub‐speciality in cardiology is now called neurocardiology.^14^ As most cases were found to be related, this research aims to provide the first data on cancer populations and focuses on fatal cardiac events in lung cancer site in the BM setting and associated survival.

Since the SEER program covers 28% of the US population, our findings are quite general. This research will affect the paradigms of BM screening, the techniques of clinical trials and the counselling of specific groups of cancer patients. In our study, the most presented organ related to BM and fatal cardiac events was the lung cancer site which comprises more than 80% of all cancer registered in SEER database. Besides, explain a similar hypothesis of non‐vascular effect on cardiac function. With different survival, the fatal cardiac events experienced a lower remarkable survival in BM history than non‐BM patients. The ischemic brain injury may play a negative predictor of survival and lead to unfavorable survival with heart‐specific patients as a novel non‐vascular cause. In one prospective clinical study, Yu et al. reported the heart variability rate is a prognostic predictor in BM patients.^15^ Such variability of rate was hypothesized to derive from autonomic impairments caused by the presence of BM, namely a non‐ischemic mechanism.

The predominant type of histology was attributed mostly to non‐small‐cell lung cancer NSCLC, which has the worse survival in patients with BM. Several reports reported the guidelines and risk factors for NSCLC BM.^16^, ^17^, ^18^, ^19^ but non‐survival inferiority and associated cardiac dysfunction were explained. In the multivariate analysis, we found the metastatic liver pattern has been associated with poorer survival of heart‐specific death in BM patients. While the radiation and chemotherapy were associated with better survival, the specific type of chemotherapy or radiation site for BM was not determined.

Our study must be taken into account in the context of the drawbacks. Next, we could classify the BM for early diagnosis of cancer. SEER does not provide details on the disease's recurrence, so we did not recognize patients who acquired BM after the initial diagnosis. For cancers that appear to be present at an early stage, this is a significant disadvantage of the database. Other studies have shown that BM continues to occur over time in existing patients with the metastatic disorder.^20^, ^21^ Second, there is no information on the volume or size of metastases present in the brain. Third, screening is not used for specific histologies in lung cancer site. As a result, BM incidence ratio is likely to underestimate the true figure in non‐screened populations. Fourth, the exact cause of cardiac death in each group of either BM or non‐BM was not detected. Furthermore, we only investigated lung cancer to pursue some consistency in the findings and have a more homogeneous population; the inclusion of patients with small‐cell tumors could jeopardize the findings, though representing a smaller proportion. Also, we explored the neurocardiology continuum through associations, which might be a good preliminary approach but no more than hypothesis‐generating.

Our analysis provides new insights, amid these limitations, into the epidemiology of BM in the United States. Data relating to the incidence of BM, the relative proportion of patients with known BM among different types of cancer, and the prognosis of patients with BM and associated fatal heart events will continue to help shape the development of screening and recommendations for care. The direct and indirect interconnections between the heart and brain injury of any cause have led to the new concept of cardiocerebral syndrome.^22^, ^23^

## 5 CONCLUSION

In cancer patients, the majority of heart‐related deaths were associated with the cancer of the lung. BM was significantly associated with lower survival of patients with heart‐specific death than non‐BM. Liver metastatic lesions were negatively associated with poor survival in BM patients. While preliminary, our findings call for further study and confirmation to understand better the processes of cardiac dysfunction in the presence of BM. Eventually, where validated, our research paves the way for personalized therapies for patients, especially to prevent, diagnose, and manage cardiovascular outcomes in the presence of BM, regardless of the presence of cardiovascular comorbidities or risk factors. We may be at the gates of a new field of scientific research on neurocardiooncology that requires further investigation of the effects of cardiac function with brain cancer lesions.

## CONFLICT OF INTEREST

None declared.

## AUTHOR CONTRIBUTIONS

Mohammed Safi: conceptualization, data curation, Formal analysis, software, writing ‐ original draft, Mohammed, Murad, Dario, and Mubarak: writing ‐ review & editing interpretation. Ravindran, Aziz, Mahmoud, Syed, and Abdullah: visualization, validation: Jiwei Liu: supervision and project administration.

## ETHICAL APPROVAL

The ethical statement is given permission to the SEER study data files by using the reference number 19916‐Nov2019.

## INFORMED CONSENT

Not applicable.

## DISCLOSURE

The abstract was accepted in European lung cancer congress ELCC 2021.

## Supporting information

Fig S1

## Data Availability

The data that support the findings of this study were derived from the following resource available in the public domain [www.seer.cancer.gov].
