# Supplementary figures and images for: Brain and heart‐specific death in cancer patients: Population‐based study
Source: Cancer Med. 2021 Aug 10;10(17):5739–47. doi: 10.1002/cam4.4069 (PMC8419745; doi:10.1002/cam4.4069)

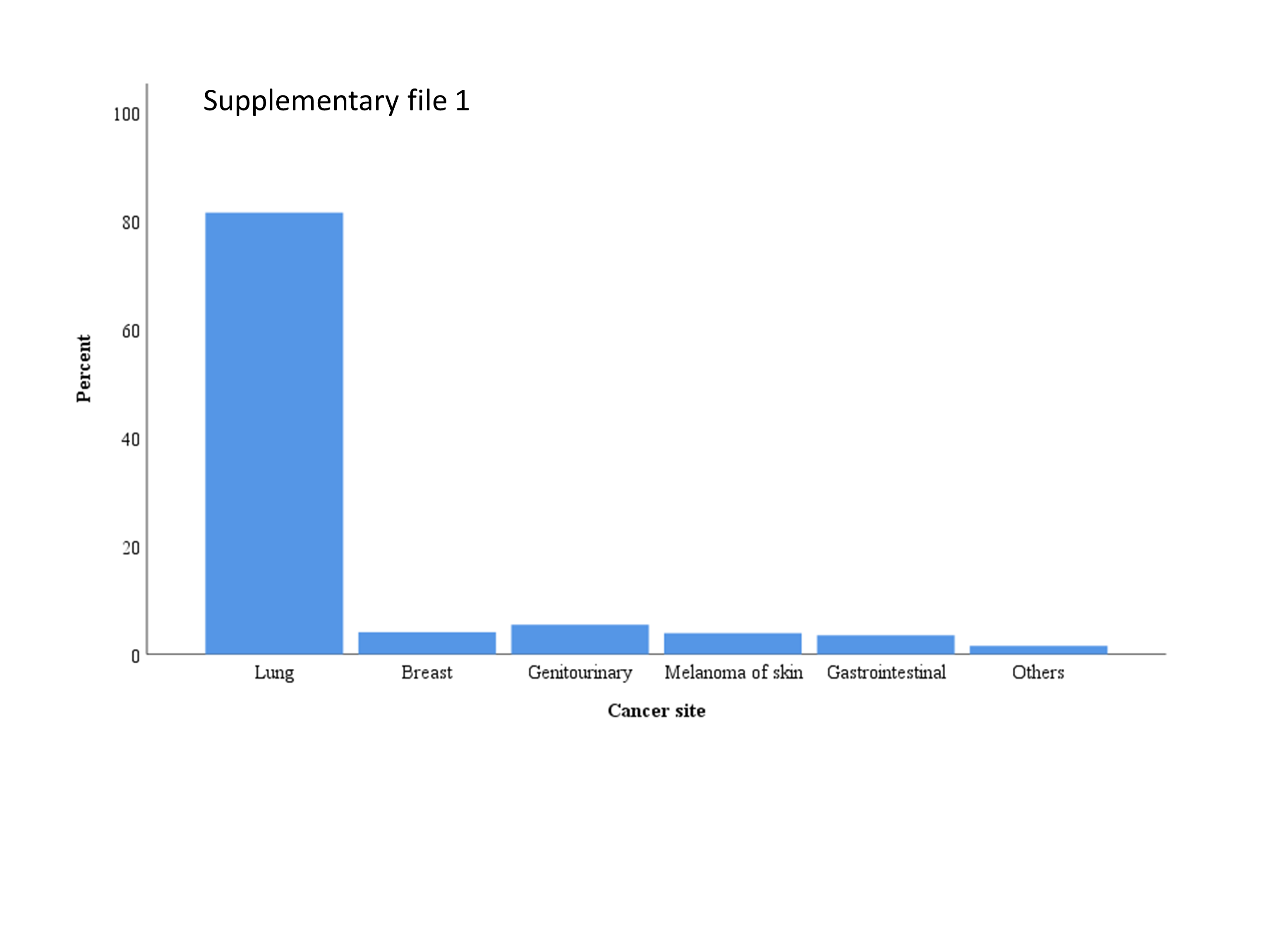

Supplement: Supplementary file 1 — Fig S1 [file CAM4-10-5739-s001.tif]
